# Sex differences in glutamate transmission and plasticity in reward related regions

**DOI:** 10.3389/fnbeh.2024.1455478

**Published:** 2024-09-18

**Authors:** Alyssa R. Kniffin, Lisa A. Briand

**Affiliations:** ^1^Department of Psychology & Neuroscience, Temple University, Philadelphia, PA, United States; ^2^Neuroscience Program, Temple University, Philadelphia, PA, United States

**Keywords:** sex differences, glutamate, long-term depression (LTD), long-term potentiation (LTP), spine density, structural plasticity, electrophysiology

## Abstract

Disruptions in glutamate homeostasis within the mesolimbic reward circuitry may play a role in the pathophysiology of various reward related disorders such as major depressive disorders, anxiety, and substance use disorders. Clear sex differences have emerged in the rates and symptom severity of these disorders which may result from differing underlying mechanisms of glutamatergic signaling. Indeed, preclinical models have begun to uncover baseline sex differences throughout the brain in glutamate transmission and synaptic plasticity. Glutamatergic synaptic strength can be assessed by looking at morphological features of glutamatergic neurons including spine size, spine density, and dendritic branching. Likewise, electrophysiology studies evaluate properties of glutamatergic neurons to provide information of their functional capacity. In combination with measures of glutamatergic transmission, synaptic plasticity can be evaluated using protocols that induce long-term potentiation or long-term depression. This review will consider preclinical rodent literature directly comparing glutamatergic transmission and plasticity in reward related regions of males and females. Additionally, we will suggest which regions are exhibiting evidence for sexually dimorphic mechanisms, convergent mechanisms, or no sex differences in glutamatergic transmission and plasticity and highlight gaps in the literature for future investigation.

## 1 Introduction

Glutamate is a major neurotransmitter in the brain that mediates fast excitatory signals. Tight regulation of glutamate transmission is required for normal cognitive functions including learning, memory, and mood regulation. Disruption in glutamate homeostasis within mesolimbic structures contributes to various reward-related psychiatric disorders, including major depressive disorder (MDD), anxiety, and substance use disorder (SUD) (Li et al., 2019; Miladinovic et al., 2015; Niedzielska-Andres et al., 2021; Gass and Foster Olive, 2008). Many of these disorders exhibit sex differences in both rates of diagnosis and symptom severity and therefore may result from sexually dimorphic pathophysiology involving glutamate (Green et al., 2019). Indeed, preclinical research suggests that sex differences within the glutamate system exist in many brain regions (Giacometti and Barker, 2020; Wickens et al., 2018). To understand potential sex differences in the role of glutamate in neuropsychiatric pathophysiology, it is important to first delineate baseline sex differences in glutamatergic transmission.

In addition to sex differences in baseline glutamatergic transmission, differences also exist in the capacity of the glutamate system to undergo structural remodeling and functional changes in synaptic strength. Synaptic restructuring is a form of plasticity resulting from acute or long-term changes in glutamatergic activity that can be analyzed by measuring morphological features such as spine density, dendritic branching, and receptor composition (Bernardinelli et al., 2014). Furthermore, glutamate plays a role in the functional strengthening or weakening of synaptic connections, which may result in long-term potentiation (LTP) and long-term depression (LTD), amongst other forms of plasticity (Citri and Malenka, 2008). Therefore, in addition to differential levels of glutamatergic transmission, baseline sex differences in synaptic plasticity may also contribute the pathophysiology of the abovementioned reward-related disorders.

This review will consider preclinical rodent literature directly comparing glutamatergic transmission and plasticity in reward related regions of males and females. This will further the understanding of how glutamate may be differentially contributing to baseline sex differences across regions. Although many other aspects of glutamatergic transmission are critical to evaluate, this review will focus on measures of structural and electrophysiological properties of synaptic plasticity in order to highlight the lack of literature focusing on sex differences in this area. The regions considered will be the nucleus accumbens (NAc), prefrontal cortex (PFC), amygdala, hippocampus, as they are key regions in the mesolimbic pathway that are implicated in reward-related disorders (Lewis et al., 2021). When possible, we will attempt to relate changes at the synaptic level to broader circuit level reward-associated behaviors. However, more work is needed to directly correlate synaptic properties with behavioral outputs. Further, it will discuss the hypothalamus and sensory regions as both regions also contribute to reward related behaviors. Additionally, we will suggest which regions are exhibiting evidence for sexually dimorphic mechanisms, convergent mechanisms, or no sex differences in glutamatergic transmission and plasticity. Lastly, we will illuminate gaps in the literature and provide suggestions for future studies to expand the field.

### 1.1 Structural and electrophysiological measures of glutamate transmission

Structural differences in glutamatergic neurons can indicate the relative synaptic strength in a specific region. Synaptic remolding is a form of structural plasticity that refers to the physical modification of neuronal networks in an activity dependent manner. Neurons adapt in response to stimuli or changes in an individuals’ environment to undergo morphological rearrangement, affecting dendritic spines and dendritic branching. Dendritic spines are highly dynamic structures that rapidly undergo changes in composition on a time scale from minutes to days (Bernardinelli et al., 2014). Variation in spine size, shape, and content have been shown to correlate with synaptic strength, maturity, and stability of glutamatergic signaling (Arellano et al., 2007; Hering and Sheng, 2001). Likewise, the extent of dendritic branching may also reflect the extent of glutamatergic connectivity in a specific region. Morphological analysis typically includes staining or fluorescently labeling neurons and using fluorescent or confocal microscopes in combination with imaging software to quantify various aspects of spine and dendrite properties (Li et al., 2023).

In addition to structural differences associated with changes in glutamatergic transmission that can be visualized in brain slices, glutamatergic transmission can be assessed using electrophysiology. With this technique, researchers can ascertain how neurons or populations of neurons communicate via glutamate. This technique is a powerful in and ex-vivo tool used to understand the state of neurons with a high level of control over the physiological environment and spatial precision. Information regarding the properties of neurons at resting states and after stimulation can be used to understand mechanisms of glutamatergic transmission in various regions of the brain. In addition to whole cell states, highly specific protocols can further delineate differences in presynaptic and post synaptic glutamate transmission and measure long term responses to stimulation. Together with structural analysis, electrophysiological properties give a more complete picture of glutamatergic transmission.

### 1.2 Synaptic plasticity

Along with differences in glutamatergic transmission, synaptic plasticity, or the ability for neurons to alter synaptic transmission in response to changes in electrochemical environment, can also be measured using slice electrophysiology. There are several forms of synaptic plasticity. The most well described forms are LTP and LTD, or the long-lasting enhancement or depression of synaptic strength over time (Abraham et al., 2019; Bear and Malenka, 1994). It is believed that this form of synaptic plasticity is the basis of long-lasting effects, including learning and memory. LTP and LTD are commonly mediated calcium influx through NMDA glutamate receptors, typically through activation of both pre- and postsynaptic sites and stabilize through the mobilization of AMPA receptors (Lüscher and Malenka, 2012; Chater and Goda, 2014). Other forms of plasticity, such as short-term plasticity, are on quicker timescales and reflect transient changes in synaptic transmission which may be critical for fast computational processes (Hennig, 2013). Many forms of short term plasticity have been recognized and play a critical role in short-term adaptations. Most forms are induced by transient changes in neurotransmitter release due to the accumulation of calcium at presynaptic terminals (Citri and Malenka, 2008; Lüscher and Malenka, 2012; Chater and Goda, 2014). To investigate differences in synaptic plasticity, stimulation protocols are used which mimic naturally occurring electrochemical environments that elicit either short or long-term changes in glutamatergic signaling. In combination with glutamatergic transmission, these measurements of synaptic plasticity give insight to a region’s ability to adapt to changing environments.

### 1.3 Nucleus accumbens

The NAc is part of the mesolimbic circuit and is widely recognized to play a critical role in reward and motivated behavior (Alcaro et al., 2007; Floresco, 2015). Composed mainly of GABAergic medial spiny neurons (MSNs) NAc receives glutamatergic inputs from the PFC, amygdala, thalamus, hippocampus, and ventral tegmental area (VTA) (Gipson et al., 2014; Li et al., 2018). Further, the NAc is subdivided into the core and shell, each with distinct contributions to motivated behavior and unique circuitry (Zahm, 1999). Understanding morphological sex differences in the NAc therefore provides insight to differences in glutamate transmission. Several sex differences have been found when directly comparing NAc dendritic morphology. Female rodents have greater spine density in the NAc core compared to the NAc core of male rodents (Forlano and Woolley, 2010; Wissman et al., 2011). A higher spine density is thought to reflect more glutamatergic input into the region with more synaptic connections. In parallel, females also have larger spines in both core and shell (Forlano and Woolley, 2010). Spine size measurements, including head diameter have been positively correlated with synaptic strength and allow for more surface area to regulate synaptic efficacy at pre- and post-synaptic levels (Arellano et al., 2007). Although most of the work suggests higher spine density and spine size in females, contrasting evidence suggests neuronal morphology in the core and shell did not differ between males and females when estrous cycle was disregarded (Beeson and Meitzen, 2023; Meitzen et al., 2011). Discrepancies in spine density and size may in part be due to differing staining and imaging techniques or due to differences in spine density on a rostral-caudal gradient or fluctuations in gonadal hormones (Wissman et al., 2012). Overall, these studies suggest that females receive more glutamatergic input and have higher synaptic transmission in the NAc than males.

As these morphological studies do not directly measure glutamatergic transition, whole-cell patch clamp electrophysiology studies are needed to determine if the structural sex differences lead to functional alterations. Consistent with the structural studies, electrophysiology findings report greater synaptic strength in female rodents compared to males. Within the NAc core, females report higher miniature excitatory post synaptic current (mEPSC) frequency (Wissman et al., 2011). Increases in mEPSC frequency could be produced either by increases in presynaptic glutamate release and/or a larger quantity of synaptic connections (Han and Stevens, 2009). This is in line higher spine density in females and with findings showing that females have larger readily releasable pools (RRP) than males (Deutschmann et al., 2022; Knouse et al., 2023). Together these measures indicate an overall higher presynaptic input in females in comparison to males in the NAc. Measures of postsynaptic strength such as mEPSC amplitude and AMPA/NMDA ratio also suggest higher glutamate transmission in females (Knouse et al., 2023). When exclusively considering properties in the NAc shell, no sex differences were found in MSN intrinsic properties or mEPSCs (Willett et al., 2016). Likewise, there are no differences between males and females on various intrinsic MSN properties when estrous cycle was disregarded or when both male and female animals are gonadectomized. However, when estrus cycle is considered, gonadal hormones seem to influence many intrinsic MSN properties suggesting that circulating hormones may contribute to many functional sex differences in this region (Proaño et al., 2018).

The NAc of females overall show more robust pre- and post-synaptic glutamatergic transmission. However, electrophysiology studies investigating synaptic plasticity are sparse and more work is needed to delineate baseline differences in short and long-term plasticity. To date, evidence suggests females may be less plastic than males in the NAc core. LTD in this region was harder to induce in wildtype females compared to males. This was concluded to be due to larger RRP and heighted glutamatergic activity in females (Knouse et al., 2023). Although there are differences in long term plasticity, no differences were found between males and females in short term plasticity, evidenced by similar evoked responses in a paired pulse paradigm (Wissman et al., 2011). To fully understand baseline sex differences in plasticity within the NAc, more studies are needed that use differing stimulation protocols to elicit a full range of responses.

Unlike most other regions in the brain, in the NAc, sex differences in glutamate transmission are consistent across neuronal morphology and electrophysiology measures. In both the core and shell, females exhibit higher presynaptic and postsynaptic markers of synaptic strength. These differences may underlie sex differences in reward-related disorders or lead females to be more vulnerable to exogenous agents that effect the mesolimbic pathway. For example, women report faster escalation from drug use to abuse and find it more difficult to quit than men, potentially due to more excitatory transmission in the NAc (Becker and Hu, 2008). Likewise, females may exhibit less plasticity in the NAc, which may contribute to an impaired ability to regulate drug seeking. However, men and women are similarly likely to become addicted to drugs of abuse (Anthony et al., 1994). It is likely that males and females are exhibiting sexually dimorphic characteristics in glutamate transmission in this region that may be contributing to differences in symptom expression in men and women.

### 1.4 Prefrontal cortex

Much like the NAc, there are clear sex differences in neuronal morphology within the PFC. These differences are not as stark as found in the NAc but underscore that the mechanisms that lead to homeostatic plasticity vary across sex. The PFC mediates higher brain functions and has been found to be incredibly plastic in response to experience (Anastasiades and Carter, 2021; Kolb and Gibb, 2015). The PFC is composed of excitatory pyramidal neurons (70–90%) and GABAergic interneurons (DeFelipe et al., 2013). When available, the distinction between the subregions will be provided with limited discussion on behavioral impacts. Very few studies have directly compared neuronal morphology between the sexes. Females exhibit higher synaptic density in both the infralimbic and prelimbic areas by measurement of immunofluorescent density of synaptophysin, a marker for presynaptic sites (Carvalho-Netto et al., 2011). However, male and female animals exhibit the same number of boutons in the PFC (Drzewiecki et al., 2016). Overall higher synaptophysin immunofluorescent density in females is therefore likely due a similar quantity but larger spines. Indeed, females show higher levels of synaptosomal GluA1 and A2 glutamatergic receptor subunits, with no differences in overall expression between the sexes (Knouse et al., 2022). Likewise, females exhibited higher mGluR5 and NR1 expression (Wang et al., 2015). Together, this indicates greater synaptic AMPA subunit expression at the synapse, potentially contributing to spine size and heightened glutamatergic transmission in females. Another distinct factor in morphological indications of glutamatergic transmission is dendritic branching. Males have a higher mean total branches and apical dendrites in layer 2/3 pyramidal neurons (Kolb and Stewart, 1991). However, this study is dated, and no study has replicated these findings. Taken together, it is possible that males and female respond differently to presynaptic input in the PFC. Females contain larger spines indicating higher synaptic strength and males may maintain a higher level of dendritic arborization, potentially serving as a homeostatic mechanism to execute similar levels of glutamatergic transmission in this region. More work needs to be done to replicate findings suggesting sex differences in dendritic branching in this region.

Functional sex differences in glutamatergic transmission in the PFC are less clear. Many neuronal properties such as resting membrane potential, rheobases, and maturational trajectories of current-voltage relationships are the same in male and female animals, indicating similar levels of glutamate signaling (Bernabeu et al., 2020; Urban and Valentino, 2017). Likewise, no sex differences in input/output relationship are reported (Bernabeu et al., 2020). This suggests that on many levels, males and females share similar basal states and respond to stimulation in the same manner. Measurements of synaptic strength are mixed. Females exhibit heightened spontaneous excitatory postsynaptic current (sEPSC) frequency, amplitude, and rectification index in comparison to male animals (Knouse et al., 2022). However, contradictory evidence shows higher sEPSC and mEPSC frequency in males with no differences in amplitude (Urban and Valentino, 2017; Pena-Bravo et al., 2019). Conflicting evidence may be due to differences between prelimbic and infralimbic subdivisions in the PFC and differences in methodological approach.

Similarly to the NAc, few studies have investigated sex differences in synaptic plasticity in the PFC. A study probing the prelimbic subregion of the PFC found no sex difference in LTP and LTD. The lack of sex differences in this study was found to be related to convergent mechanisms. When probed deeper, it was revealed that a different set of receptors were employed between the sexes to produce similar LTD, suggesting sexually dimorphic mechanisms at play (Bernabeu et al., 2020). It is likely that this is true for many regions throughout the brain as steroid hormones are known neuroplasticity modulators. However, more studies evaluating the role of biological sex on synaptic plasticity are needed.

In the PFC, subtle sex differences in neuronal morphology serve as a prime example of how males and female may vary in mechanism but produce similar output. While females have larger spines in the PFC, it is possible that males have more dendritic branching, although this finding has yet to have been replicated. Functional studies utilizing slice electrophysiology are mixed. It is possible that at a resting state, functional outputs are similar, but females may have the machinery in place to have more flexibility than males and more readily response to changes in stimuli due to morphological differences. The PFC is critical for cognitive flexibility and decision making and therefore is involved in reward and aversion based learning (Pastor and Medina, 2021). Further, the connection between the PFC and the NAc is heavily involved in many aspects of the drug addiction cycle and is related to both reward-seeking and impulsivity (Perry et al., 2011). Women, for example, report greater craving induced by cues and may contribute to a greater vulnerability to drugs of abuse (Fox et al., 2013). It is possible that differences in mechanisms in glutamatergic transmission between the sexes contributes to sex differences found in these behaviors.

### 1.5 Amygdala

The amygdala is the central region associated with emotional regulation and plays a major role in the mesolimbic reward pathway. Unlike the NAc and PFC, the influence of biological sex is less clear. This lack of clarity may be due, in part, to the many distinct nuclei within the amygdala which may not all contain the same morphological features. Males contain a higher number of dendritic shaft synapses in the amygdala compared to females in the medial nucleus (Nishizuka and Arai, 1981). Similarly, males have a higher spine density than females in the basolateral amygdala (BLA) (Rubinow et al., 2009). However, contradictory evidence suggests that females have more presynaptic sites than males in the central, basolateral, and medial amygdala compared to males (Carvalho-Netto et al., 2011). Likewise, females contain more spines in the basal and lateral nucleus and have a higher density of GluR1 expression in the lateral amygdala compared to males (Carvalho-Netto et al., 2011; Blume et al., 2017). The studies that conclude females to have heightened glutamatergic transmission are more compelling due to technical aspects and a wider array of methods and nuclei under analysis. Although there is stronger evidence for heighted glutamatergic transmission in females, no sex differences in the number of neurons in the amygdala or amount of dendritic branching between the sexes (Gass and Foster Olive, 2008; Pena-Bravo et al., 2019; Pastor and Medina, 2021; Arpini et al., 2010). Altogether, inconsistencies across studies may instead suggest differing underlying mechanisms maintaining glutamatergic transmission in this region, potentially due to the heterogeneity of cells in the amygdala. More detailed work including both sexes throughout the amygdala is needed to better understand this region.

Unfortunately, very little work has been done to understand sex differences in physiology within the amygdala. A whole-cell patch clamp electrophysiology study reports higher firing rate and higher mEPSC frequency and amplitude in female animals compared to male animals in the BLA (Blume et al., 2017). This study also reported female tissue used glutamate more effectively than males when using iontophoretic glutamate application techniques (Blume et al., 2017). However, the opposite was found in the posterior division of the medial nucleus of the amygdala, showing that males had a higher firing rate than females (Dalpian et al., 2019). While the BLA contributes to reward, the medial division of the amygdala is involved in copulatory behaviors. Here, differential levels of excitatory transmission are expected due to necessary sex specific copulatory behaviors. However, not all electrophysiological studies have revealed sex differences, Corbett et al. (2023) reports no difference between male and female animals in sEPSC frequency and sEPSC amplitude. Likewise, no differences in passive membrane properties of these cells were found between the sexes (Dalpian et al., 2019). Perhaps, differing nuclei containing stronger or weaker connectivity may balance one another and lead to overall similar glutamatergic transmission across the region. This would suggest that males and females have similar overall functional output but have differing patterns of connectivity that lead to sex specific behavioral outputs.

Few studies have looked at sex differences in synaptic plasticity in the amygdala. Synaptic plasticity in the BLA plays a role in rodent behaviors such as freezing and components of fear conditioning (Maren and Fanselow, 1995). Female rats exhibit heightened levels of both cued fear freezing behavior and LTP in the LA. The lower levels of LTP and freezing are mediated by testosterone, whereas ovarian hormones are loosely associated with heighted LTP and freezing in females (Chen et al., 2014). However, this is the only study found to investigate sex differences in synaptic plasticity, and similarly to other regions, more is needed to understand the differences in plasticity and the potential contribution to differences in behavior.

The amygdala plays a key role in integrating information from various sources and contributes to emotion, learning and memory, reward, and motivation (Murray, 2007). Transient glutamatergic activity in the BLA, for example, is time locked to reward seeking in rats (Malvaez et al., 2015). Hyperactivity in the amygdala is linked to susceptibility of stress-related psychiatric disorders due to the close parallel regulation of the hypothalamic pituitary adrenal (HPA) axis. Stress related psychiatric disorders are more prevalent in women. Many different functional sex differences have been examined in the amygdala in human populations. For example, women have enhanced amygdala responding during aversive stimuli and exhibit more negative emotions (Domes et al., 2010; Stevens and Hamann, 2012). This may be partially explained by heightened glutamatergic transmission in females and greater synaptic plasticity. However, this is based on very few studies that do not all align with this conclusion. Additionally, sex differences men and women could be explained by differences in areas of recruitment during the evaluation of emotional stimuli, and less so explained by baseline sex differences in glutamatergic transmission and plasticity.

### 1.6 Hippocampus

Decades of research has provided substantial information on hippocampal function and circuitry (Amaral and Witter, 1989; Chauhan et al., 2021). Clear differences have emerged in glutamatergic tone between males and females. Females have a greater spine density in the CA1 region and higher expression of glutamate receptor subunits such as mGluR2, mGluR3, mGluR5, NR2B compared to males (Wang et al., 2015). Females also have larger spines along dendrites of pyramidal neurons and higher levels of NMDA1 and NMDAR2 receptor expression (Brandt et al., 2020). However, no differences were found in the total number of synapses between mossy fibers and apical dendrites (Madeira et al., 1991). In opposition to the sex difference in spine morphology, male neurons in the CA3 region have longer dendrites and more dendritic volume than females (Isgor and Sengelaub, 2003). Together, this suggests although females may have a higher level of structural connectivity and glutamatergic transmission and that males may compensate this difference by having more advanced dendritic arborization in the CA1 and CA3 subregions. In other subregions of the hippocampus, no sex differences are found. A study looking specifically at the dentate gyrus found no sex differences in spine volume or density (Niiyama et al., 2020), an effect that may be specific to the dentate gyrus. Therefore, sex differences within the hippocampus in spine morphology and glutamatergic transmission may be subregion specific.

The sex differences in electrophysiology properties of glutamatergic neurons in the hippocampus suggest greater transmission in male animals. Males exhibited a higher input-output curve in response to increasing stimulus intensity while also showing greater activity at excitatory synapses than females (Harte-Hargrove et al., 2015; Sertel et al., 2021). This robust effect can be seen in fEPSC slope, amplitude, and parallels a sex difference in NMDA receptor activation and more efficient use of vesicle pool recycling and stronger local translation at the synapse (Maren, 1995; Monfort et al., 2015). However, not all neuronal properties are stronger in males. Under basal conditions, females showed larger AMPA receptor-mediated synaptic response (Monfort et al., 2015), and in cultured neurons, females presynaptic terminals exhibited a higher number of synaptic vesicles compared to males (Kim et al., 2023). Here, no sex differences were found in presynaptic protein expression, vesicle endo- or exocytosis, or presynaptic calcium alternation (Kim et al., 2023). Although not all parameters of glutamatergic transmission are greater in males, most studies suggest heighted glutamatergic transmission in male rodents.

The hippocampus was the first region used to understand synaptic plasticity, with major focus in delineating mechanisms for learning and storage of memory (Avchalumov and Mandyam, 2021). To date, much of this work was exclusively done in male animals. With the recent inclusion of female animals, it is clear that sex differences in synaptic plasticity exist. Across varying electrophysiology protocols, male animals exhibit a higher magnitude of LTP in the CA1 region and the perforant pathway-dentate gyrus synapse (Sertel et al., 2021; Maren, 1995; Monfort et al., 2015; Safari et al., 2021). Likewise, male animals can respond to a broader range of tetanic stimuli for the induction of LTP compared to females. However, further investigation studying mechanisms underlying this plasticity are sexually dimorphic. Females and males have differences in mechanisms and thresholds for field CA1 LTP, with different kinase activation and NMDA receptor association (Wang et al., 2018). LTP in males requires NMDARs while LTP in females occurs independent to NMDAR activation. Additionally, estradiol induced LTP recruits a different set of kinases between the sexes, with only females requiring cAMP-activated protein kinases. Females were found to utilize both L-type calcium channels and internal calcium stores whereas in males, either resource is sufficient to permit potentiation (Jain et al., 2019). These studies are some of the first to show mechanistically different processes that produce the same endpoint. It is possible therefore that many regions may share these sexually dimorphic synaptic plasticity mechanisms that have yet to be determined.

### 1.7 Hypothalamus

Very few regions of the brain aside from the mesolimbic circuitry have been investigated for sex differences in glutamate transmission and plasticity. Outside of the traditional mesolimbic circuitry, the hypothalamus is critical for the processing of basic or primary rewards (ScienceDirect, 2013). The hypothalamus is the most well documented due to its involvement in endocrine and autonomic nervous systems that regulate reproductive behaviors, appetite, motivational states, energy balance and circadian rhythms (Schröder et al., 2020). Clear sex differences in this region have been found, specifically relating to the larger size of the sexually dimorphic nucleus of the preoptic area (SDN-POA) in males compared to females (Gorski et al., 1978). However, subtle sex differences in spine morphology can be found throughout the hypothalamus (van den Pol and Trombley, 1993). Male animals contain larger and more synaptically dense hypothalamic nuclei than females. The ventral-medial hypothalamus in male rodents is approximately 1.25 times larger than females (Dugger et al., 2007; Dulce Madeira et al., 2001). Likewise, males display a higher number of axo-spinous synapses and spine density in the ventral-lateral and ventromedial hypothalamus (Larriva-Sahd et al., 1995; Matsumoto and Arai, 2008; Pozzo Miller and Aoki, 1991), with only one study reporting the opposite finding (Dulce Madeira et al., 2001). Higher spine density and more synaptic connections suggests higher glutamatergic transmission in males in the hypothalamus. However, this may not be uniform across all subregions in the hypothalamus, for example females exhibited higher presynaptic markers in the paraventricular nucleus of the hypothalamus (Carvalho-Netto et al., 2011).

Thus far, no work has been done to delineate sex differences in synaptic plasticity within the hypothalamus. The hypothalamus is part of the HPA axis and extensive literature has found sex differences this region in response to stress (Heck and Handa, 2019). These sex differences exist in hormonal responses to stress, in feedback mechanisms, and stress induced receptor mobilization. Varying neuroendocrine responses contribute to sex differences in stress-related psychiatric disorders. Less work has been done to elucidate the influence of biological sex on glutamatergic transmission and how these differences may contribute to the overall sex differences in behavior.

### 1.8 Sensory cortices

Very little work has investigated sex differences in other brain regions besides the abovementioned. Therefore, this discussion will combine available literature on sensory regions. In the visual cortex, Females report lower spine density due to fewer stubby and mushroom spines (Parker et al., 2020). Similarly, young adult male rats display a higher dendritic tree and spine density in the anterior cingulate cortex in comparison to females (Markham and Juraska, 2002). Although limited, it is possible that there is a pattern higher glutamatergic transmission in females, evidenced by higher structural plasticity in these regions.

Studies utilizing electrophysiology in cortical regions to understand functional differences in synaptic plasticity generally report a lack of robust sex differences. No sex differences were found in the magnitude of LTP within the anterior cingulate cortex or the recruitment of silent synapses (Liu et al., 2020). Likewise, males and female neurons do not differ in active or passive membrane properties in the insular cortex (Iezzi et al., 2023). Although there are no differences in measures of LTP in the anterior cingulate cortex, LFS was more likely to induce LTD in male mice in comparison to females. This subtle difference implies a sex difference in network plasticity (Iezzi et al., 2023). This work highlights that although males exhibit higher baseline structural plasticity markers, most neuronal properties are similar in males and females throughout these regions. Limited studies have investigated the functional consequences for differences in glutamatergic transmission in these regions therefore more work is needed to investigate properties of glutamatergic transmission that may differ between the sexes.

### 1.9 Sex hormones and glutamate transmission

Sex hormones have been well documented to influence aspects of glutamatergic transmission (Arevalo et al., 2015; Kuwahara et al., 2021). Evidence from studies investigating differences in cognition and synaptic plasticity between phases of the estrous cycle demonstrate how fluctuation in female hormones can modulate plasticity (Proaño et al., 2018; Warren et al., 1995; Woolley et al., 1990). Although full discussion is outside the scope of this review, we will briefly consider the impact of estrogens and androgens on structural and functional plasticity. Generally, spine density is increased with the administration of sex hormones. Spine density increased after the administration of estrogens in regions such as the PFC, hippocampus, hypothalamus and somatosensory cortex (Garza-Meilandt et al., 2006; Calizo and Flanagan-Cato, 2000; Segarra and McEwen, 2008). Similarly to estrogens, sub chronic administration of androgens increased dendritic spine density in the PFC and hippocampus in female rats (Luine et al., 2022; Leranth et al., 2004) and in the hippocampus of male rats (MacLusky et al., 2004).

Electrophysiology studies also confirm the ability of sex hormones to alter synaptic connections (Kuwahara et al., 2021; Brinton, 2009; Goyette et al., 2023). The removal of gonadal hormones results in decreased synaptic efficacy in the hippocampus in male and female animals (Sakata et al., 2000; Chiaia et al., 1983; Smith et al., 2002). Application of testosterone and estradiol in the hippocampus have been found to facilitate neuronal excitability (Yokomaku et al., 2003; Pettorossi et al., 2013). Estrogens potentiate presynaptic function by upregulating glutamate release (Yokomaku et al., 2003). Additionally, estrogen participates in modulation of synaptic plasticity through postsynaptic functions (Bendis et al., 2024) (see Bendis et al., 2024 for review). Less is known regarding the role of progesterone in glutamatergic synaptic efficacy however, some studies report inhibitory effects on glutamatergic transmission through actions with GABA and glutamate (Smith et al., 1987). Sex hormones modulate glutamatergic activity, however more work is needed to understand the roles of androgens and progesterone is this area.

## 2 Conclusion

**FIGURE 1:**
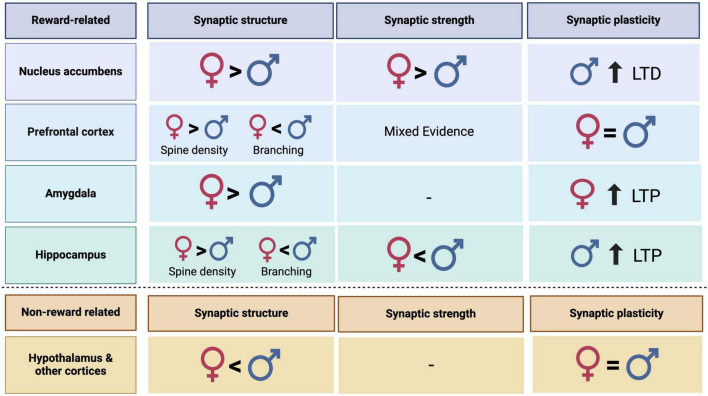
Summary table representing sex differences in synaptic structure, strength, and plasticity in reward related and non-reward related regions in male and female rodents. Dashes represent categories where more research is needed to make conclusions.

Here, we synthesized data on glutamatergic transmission and synaptic plasticity from male and female rodents, both within reward related regions to understand the influence of biological sex on reward related circuitry. We found that sex differences in glutamatergic transmission and synaptic plasticity are not uniform across the brain or even within the reward circuit. Instead, evidence for convergent and divergent mechanisms underlying these differences exists between the sexes. Although the limited availability of data on synaptic plasticity throughout the brain makes it difficult to capture the complete picture, glutamate signaling differences exist in many regions. In some cases, the differences may “cancel each other out” leading to similar levels of glutamate transmission (functional convergence) whereas others may result in sex differences (functional divergence; Figure 1).

In this first category, the functional convergence often results from mechanistic sex differences that produce similar levels of synaptic plasticity across biological sex. This may be the case for many regions beyond those discussed here but there is a dearth of published work examining these potential convergent mechanisms. The PFC falls into this category with similar levels of glutamatergic transmission across biological sex that exist on a background of sex differences within the glutamate signaling system. While females exhibit a higher number of dendritic spines, males recruit more efficient receptors, possibly contributing to similar levels of LTP and LTD. This serves as a prime example of a region that maintains consistent levels of glutamatergic signaling via different mechanisms. The PFC plays a central role in an individual’s experience of reward-related disorders such as SUD, largely controlling impulsive, motivated, and relapse behaviors (Renard et al., 2017). When developing treatments, subtle differences in glutamatergic transmission mechanisms between the sexes are paramount. Although the region may display similar levels of glutamate transmission, targeting a specific aspect may necessarily have to be sex specific. Continuing to investigate the mechanisms underlying similar levels of synaptic plasticity between the sexes is therefore critical to the ongoing development of neurological treatments.

Unlike the PFC, which may display converging mechanisms and similar levels of glutamatergic transmission between the sexes, other regions such as the hippocampus and the NAc exhibit sexually dimorphic mechanisms. The NAc shows higher glutamatergic transmission and synaptic plasticity in females. This true baseline sex difference is the strongest out of the regions discussed and therefore be considered heavily when translating findings to human populations. The hippocampus similarly exhibits sexually dimorphic mechanisms of glutamatergic transmission and synaptic plasticity that instead favors male animals. As this region is well studied for physiological properties, divergence in mechanism can be attributed partially due to hormonal modulation and differential receptor recruitment. Continuing to delineate mechanist differences in glutamate transmission within both the NAc and the hippocampus may provide insight to sex specific treatments or understanding of specific vulnerability.

Still other regions of the brain have limited research on the influence of biological sex in glutamatergic signaling. While the hypothalamus and other regions display evidence for morphological markers of higher synaptic plasticity in males, there is a lack of functional data to support this claim. Lack of clear sex differences in these regions may represent little to no influence of biological sex on glutamatergic transmission. Discrepancies in sub regions or in methodological approaches may contribute to inconsistencies in reports. However, literature purposefully comparing male and female animals on levels of synaptic plasticity across the brain are sparse. In combination across all regions discussed, articles containing direct comparison of males and females on levels of structural analysis included only 30 articles; functional measures of glutamatergic transmission were limited to 20 articles. Further, when considering a major mechanism of synaptic plasticity, LTP, only 10 articles discuss sex differences. Uncovering sex differences in these key features of glutamate transmission and synaptic plasticity is crucial for the understanding of the brain across reward and non-reward related regions. Therefore, it is clear there is much more work to be done in this space to full elucidate the influence of biological sex on glutamate transmission and synaptic plasticity throughout the brain.

Understanding sex differences in baseline levels of glutamatergic transmission may be critical for the development of treatments that target this system. For example, chronic stress may lead to cases of depression and anxiety in which there are alterations in synaptic plasticity. In the PFC and hippocampus, this results in deficits in LTP and facilitation in LTD (Marsden, 2013). To treat males and females with the highest efficacy, understanding the different mechanism that may be underlying baseline synaptic plasticity is necessary. With the increasing specificity of drug development, it may be possible to target region specific transmission and therefore, mechanistic differences between males and females will need to be understood. Likewise, treatments that may target overall glutamatergic transmission may need to consider baseline differences between males and females. A general increase in glutamate function may have drastically different effects between the sexes and have differential associated risks due to different starting points. However, by continuing to investigate the glutamatergic system and biological sex differences, we can attempt to identify targets that may benefit both men and women.
